# Effect of Plasma Excitation Power on the SiOxCyHz/TiOx Nanocomposite

**DOI:** 10.3390/mi14071463

**Published:** 2023-07-21

**Authors:** Tsegaye Gashaw Getnet, Nilson C. Cruz, Elidiane Cipriano Rangel

**Affiliations:** 1 Laboratory of Technological Plasmas, Institute of Science and Technology, São Paulo State University (UNESP), Sorocaba 18087-180, SP, Brazilnilson.cruz@unesp.br (N.C.C.); 2 Department of Chemistry, College of Science, Bahir Dar University, Bahir Dar P.O. Box 79, Ethiopia

**Keywords:** PECVD, HMDSO, plasma, TiO_2_ nanoparticle

## Abstract

Titanium dioxide has attracted a great deal of attention in the field of environmental purification due to its photocatalytic activity under ultraviolet light. Photocatalytic efficiency and the energy required to initiate the process remain the drawbacks that hinder the widespread adoption of the process. Consistently with this, it is proposed here the polymerization of hexamethyldisiloxane fragments simultaneously to TiO_2_ sputtering for the production of thin films in low-pressure plasma. The effect of plasma excitation power on the molecular structure and chemical composition of the films was evaluated by infrared spectroscopy. Wettability and surface energy were assessed by a sessile drop technique, using deionized water and diiodomethane. The morphology and elemental composition of the films were determined using scanning electron microscopy and energy dispersive spectroscopy, respectively. The thickness and roughness of the resulting films were measured using profilometry. Organosilicon-to-silica films, with different properties, were deposited by combining both deposition processes. Titanium was detected from the structures fabricated by the hybrid method. It has been observed that the proportion of titanium and particles incorporated into silicon-based matrices depends on the plasma excitation power. In general, a decrease in film thickness with increasing power has been observed. The presence of Ti in the plasma atmosphere alters the plasma deposition mechanism, affecting film deposition rate, roughness, and wettability. An interpretation of the excitation power dependence on the plasma activation level and sputtering yield is proposed. The methodology developed here will encourage researchers to create TiO_2_ films on a range of substrates for their prospective use as sensor electrodes, water and air purification systems, and biocompatible materials.

## 1. Introduction

Titanium dioxide, TiO_2_, has attracted great attention in the fields of environmental depollution [1], solar energy cells [2], photocatalysis [3,4], chemical- and bio-sensors [5], gas sensors [6,7], photoelectrodes [8,9] and electronic devices [10]. For decades, there has been special research on TiO_2_ catalytic applications regarding the elimination of environmental pollutants and, more recently, for photocatalytic processes concerning the degradation of pollutants in the air, water, and soil [11,12,13,14]. All these applications are ascribed to the excellent properties of TiO_2_, such as high production of hydroxyl radicals, low cost, non-toxicity, physical and chemical stability, and recyclability, aside from it being relatively simple to produce and forming nanostructures more readily than other catalysts. In addition, it has been widely applied because of its environmentally benign properties and self-cleaning effects [13].

The recombination of electrons and holes, ease of agglomeration during synthesis, and requirement for activation energy in the ultraviolet (UV) region of the spectrum are just a few of the limits on the effectiveness of the TiO_2_ photocatalytic process [15]. The high refractive index of anatase TiO_2_ causes high reflection and reduces visible light transmission. In addition, the anatase TiO_2_ thin films can be easily transformed into the rutile phase, which enhances the photocatalytic activity after the annealing process (~700 °C) [16,17]. Nevertheless, many types of research have focused on improving the properties of TiO_2_ concerning photocatalytic reactivity, by doping it with transition metals [18] and rare metals [19]. Although transition-metal-doped TiO_2_ showed higher photocatalytic activity than pure TiO_2_, the toxicity or hazard of transition metals is an environmental and health concern. Therefore, the development of this TiO_2_ photocatalyst, using less toxic dopants, becomes a more interesting subject. It has been reported that SiO_2_ groups can enhance the photocatalytic reactivity of TiO_2_ [20] and speed up the photocatalytic process [21,22]. In addition to having high thermal stability, excellent mechanical strength, and contributing to the formation of new catalytic centers by the interaction of TiO_2_ and SiO_2_ [23], SiO_2_ acts as a support for TiO_2_, and provides a large surface area and porous structure [24]. Moreover, similar to the case of electrode sensors, the deposition of TiO_2_ on porous substrates such as graphene, clay materials, and amorphous BiFeO_3_, is associated with photocatalytic processing of evolving impurities [25], anomalous separation of photoinduced charges [26], and potential applications in energy conversion [27].

Nowadays, TiO_2_ nanoparticles incorporated in ordered 2D hexagonal mesoporous silica (SiO_2_) films are fabricated to be used as a catalytic nanoreactor. Due to their good resistance to alkaline solutions, SiO_2_-TiO_2_ coatings attract the attention of many scientists. Many methods, such as sol-gel, chemical vapor deposition, and physical vapor deposition, have been proposed to immobilize TiO_2_ in suitable thin films [28], but plasma polymerization has emerged as a potential tool for the fabrication of organic or hybrid films with tunable performance properties to support photocatalysis. By varying the plasma excitation and adjusting the specific plasma discharge conditions, films with desired chemical structures can be obtained [29]. Therefore, it is possible to extend this technology to the aforementioned applications.

Recently, there has been an interest in using plasma polymers as a support for TiO_2_ particles, to improve the performance of the composites as chemical, physical and biological sensors, and nonlinear optical and microelectronic devices [30,31,32]. Particularly, the flexible organosilicon plasma polymers are quite interesting for sensor applications. Considering such facts, the present work was focused on the incorporation of TiO_2_ particles in organosilicon thin film structure by using a new hybrid methodology that associates the plasma sputtering of TiO_2_ powder together with plasma polymerization from hexamethyldisiloxane, HMDSO.

## 2. Experimental

**Figure 1:**
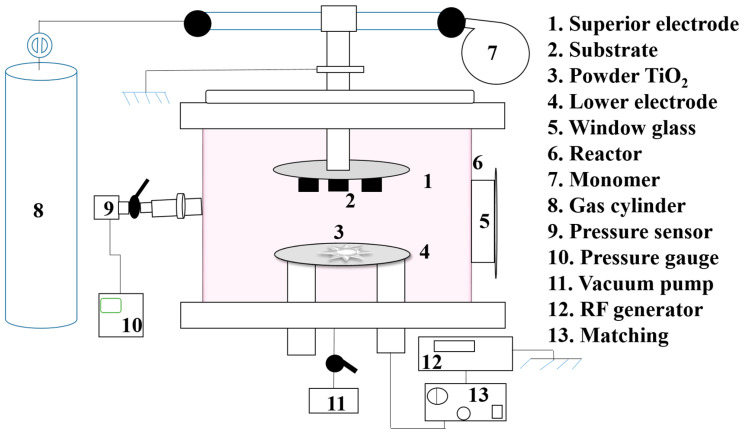
Schematic representation of the experimental setup used for film deposition.

The plasma deposition process was performed in a capacitively coupled stainless steel reactor, as shown in Figure 1. A full description of this system can be found elsewhere [33], but it essentially consists of a stainless steel reactor, a vacuum system, and a generator.

Before the depositions, polished aluminum plates and glass substrates were cleaned in ultrasonic baths for 480 s by using a detergent solution, distilled water, and isopropyl alcohol, respectively, and were finally dried with a hot air gun. Double-sided tape was used to attach the cleaned substrates to the plasma reactor’s upper electrode, while the bottom electrode received 0.8 g of titanium dioxide powder directly. The system was pumped to the background pressure (1.7 Pa) before adjustment of the pressure of the compound. The deposition process was conducted for 3600 s in radio-frequency plasmas (13.56 MHz) composed of 80% Ar (3.2 Pa) and 20% HMDSO (0.8 Pa) at a fixed pressure of 4.0 Pa, producing an overall working pressure of 5.7 Pa. The plasma excitation power varied from 50 to 200 W in five different procedures.

The chemical structure of the films prepared on polished aluminum plates was determined by infrared reflectance absorption spectroscopy. The surface morphology was examined by a JEOL (JSM-6010LA, JEOL Ltd., Peabody, MA, USA) scanning electron microscope (SEM) with a magnification of 950 and a beam energy of 3.0 kV. To avoid charging effects during the inspections, the samples were coated with a thin conductive layer obtained by Ar sputtering (30 mA, 7 Pa, 60 s) with a gold–palladium target on a Denton Vacuum HP Desk V system. The elemental composition of the films was determined by energy dispersive spectroscopy (EDS) using a Dry SD Hyper-Detector (EX-94410T1L11, JEOL Ltd., Peabody, MA, USA) interfaced with the SEM. The beam energy and spot size used in such experiments were 10.0 kV and 70 nm, respectively. Film thickness and roughness were measured using a profilometer (Veeco, Dektak 150, Veeco Metrology, Tucson, AZ, USA). To measure thickness, films were deposited on glass slides partially masked with Kapton tape (5413, 3M), which protected half of the surface from deposits. The height of the steps corresponded to the film thickness. For each sample, the thickness was measured at least 10 times in different regions of the step, using a scan length of 650 μm and a normal force of 3.0 mg. Average roughness was determined from topography profiles recorded in the step-free area sample with a scan length of 1200 μm. The wettability and surface energy of films deposited on glass slides were analyzed by the sessile drop method using a goniometer (image programs Ramé-Hart 100 and RHI 2001). To conduct the test, 3.0 μL of deionized water and diiodomethane were spotted on each sample at three different locations, and they were measured under room temperature and controlled humidity conditions.

## 3. Results and Discussion

### 3.1. Thickness and Deposition Rate

Titanium dioxide nanoparticles are widely used in cosmetics and biomedical applications such as biosensors, cancer treatment products, and drug delivery systems due to their distinctive antioxidant, antibacterial, antifungal, anticancer, antibiofilm, and photocatalytic properties. There are presently environmental and energy uses for the indiscernible film-like nano-TiO_2_ photocatalyst coating, including CO_2_ capture, air and water purification, gas detection, and renewable fuel production from water splitting.

**Figure 2:**
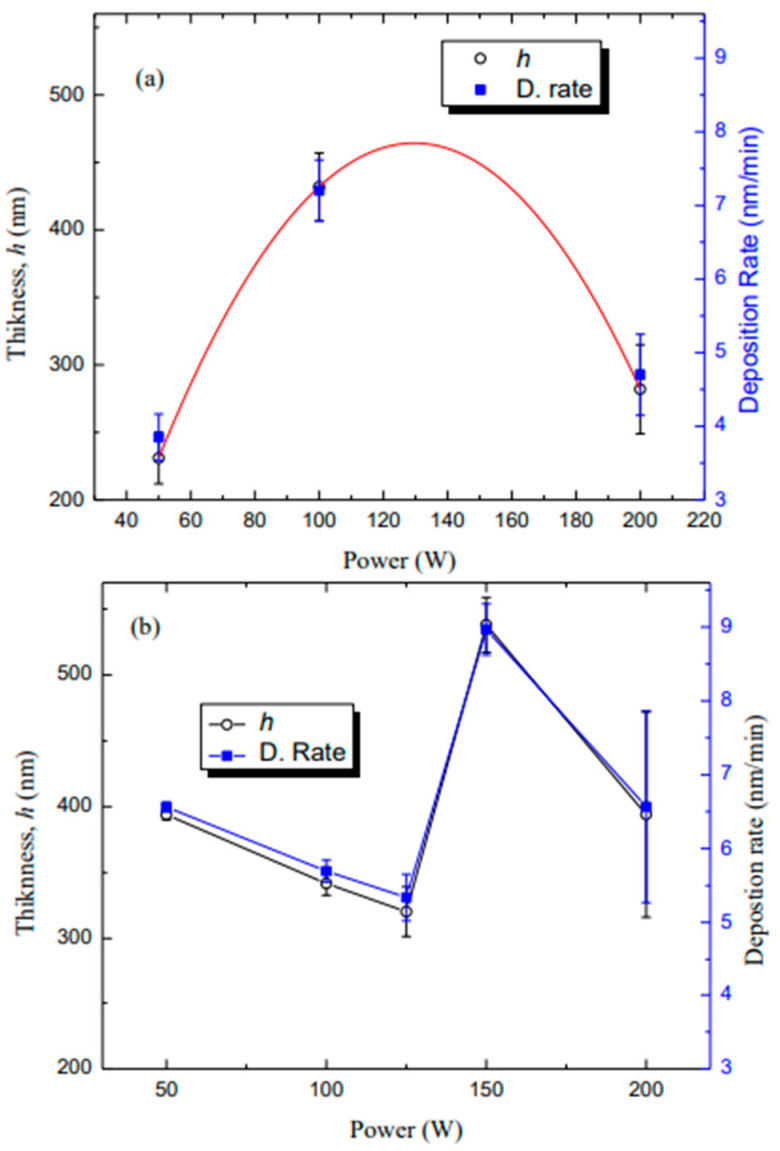
Thickness and deposition rate of (**a**) HMDSO and (**b**) HMDSO with TiO_2_ films, as a function of plasma excitation power.

Figure 2 shows the thickness, *h*, and deposition rate of the films, prepared from an HMDSO and Ar atmosphere with and without the presence of TiO_2_ powder_,_ as a function of the plasma excitation power. As can be observed, films with *h* ranging from 231 to 600 nm were obtained in these experiments with and without titanium dioxide sputtering. Considering first the experiments conducted with no TiO_2_ powder in the reactor, there is a progressive rise in *h* from 231 to 341 nm with increasing *P* up to 100 W. Afterwards, it suddenly falls to 282 nm for a further rising in power. Since deposition time, *t*, was kept constant in all the experiments, deposition rate (*h*/*t*) followed the same trend of thickness ranging from 3.9 to 7.2 nm/min.

In the principle of plasma deposition, for a low level of energy input, the extent of ionization is low and ion-molecule reactions dominate polymer formation, resulting in a deposition rate and *h* increase with increasing power until the optimum power. However, further increasing power above optimum *P* results in decreasing *h*, as well as in the rate of deposition, which can be explained by an intensive fragmentation of the HMDSO molecule, generating volatile non-sticking species, which are removed by the vacuum system. In addition, above certain levels of energy, there is a greater loss of functional groups from the monomer, resulting in competitive polymerization and ablation.

Considering the combined effect of *P* and TiO_2_ sputtering, the thickness decreased from 400 to 300 nm as *P* increased from 50 to 125 W. With further increasing *P* to 150 W promotes a sudden increase in *h*, but a new fall trend appears for the highest *P* level regime (>150 W) (Figure 2b). Since deposition time, *t*, was kept constant (60 min) in all the experiments, deposition rate (*h*/*t*) followed the same trend of thickness, ranging from 5.34 to 8.97 nm/min for a film deposition with TiO_2_ powder.

Films possessing 430 nm were reported by Vendemiatti et al. [34] using low-pressure radio-frequency plasmas (24 Pa, 150 W) composed of 70% HMDSO and 30% Ar. However, as the deposition time was 30 min in the mentioned work, the deposition rate is around twice that observed here. The incorporation of increasing proportions of oxygen in this atmosphere accelerated the deposition rate, indicating that the deposition occurred at the “lack of energy regime” under such conditions. However, higher deposition rates (33 nm/min) were attained [35] on films deposited in 70% HMDSO, 10% Ar, and 20% O_2_ plasmas in the low power regime (50 W) whereas detachment of the coatings was detected as increasing *P* beyond 75 W. Three factors are pointed to as the reason for the discrepancies: (i) in the mentioned works, high proportions of the organosilicon compound were used creating a lack of energy regime under low power levels, fastening deposition rate; (ii) the incorporation of small proportions of O_2_ accounted for increasing the activation degree of the precursor compound; and (iii) the deposition was conducted under the driven electrode for the action of extra energy provided by ion bombardment.

Thus, the overall moderate deposition rates observed here are ascribed to the high proportion of Ar present in the plasma phase, producing a lack of monomer regime together with competition amongst deposition and ablation processes. Furthermore, the sample holder is grounded and does not attract ionized fragments, as occurs when a deposition is performed on the driven electrode, slowing down the deposition rate. Finally, various reactions can take place during plasma polymerization, including excitation, ionization, hemolytic bond splitting, molecular fragmentation, etc. For a low level of energy input, the extent of ionization is low, making ion–molecule reactions dominant for polymer formation, and resulting in enhancement of the deposition rate with power. As in the present case, the deposition rate decreased with increasing *p*, which further confirms a “lack of monomer regime”. In such a regime, the film is formed mostly through reactions on the surface of the substrate, rather than on the gas phase. Owing to that, increasing the applied power may result in the deposition of smaller fragments and the sputtering of deposited species as well, both processes contributing to the decrease of the deposition rate. Furthermore, the presence of TiO_2_ powder may account for this result, since it may affect the overall plasma pressure, altering the average energy and reactivity of the plasma species.

### 3.2. Molecular Structure

**Figure 3:**
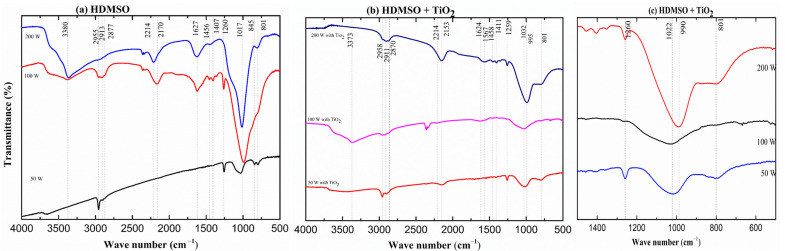
Infrared spectra of films deposited at different powers of, (**a**) HMDSO, (**b**) HMDSO with TiO_2_ sputtering, and (**c**) the same as (**b**), highlighting the low wavenumber 500–1500 cm^−1^ region.

Figure 3a shows the infrared spectra of HMDSO films under different plasma excitation powers, *P.* In these cases, the TiO_2_ precursor was not present in the deposition procedures. The spectrum of the film deposited at the lowest power regime exhibits several characteristic peaks, as in polydimethylsiloxane (PDMS) structure: asymmetric (2955 cm^−1^) and symmetric (2905 cm^−1^) CH_3_ stretching, and bending of CH_3_ (1260 cm^−1^) in Si-(CH_3_)_x_, with x = 1, 2, 3 [36]. Other most prominent absorption bands at 1032 cm^−1^ can be assigned to Si–O stretching vibration in Si-O-Si, while the peak at 845 cm^−1^ and 800 cm^−1^, is caused by methyl rocking vibration in Si(CH_3_)_3_ and Si(CH_3_) Si(CH_3_)_2_ groups, respectively (Table 1) [36,37,38]. Taking into account the above results, it can be concluded that the HMDSO and Ar atmosphere films deposited at lower *P* retain almost all characteristics of the monomer.

It can also be seen in Figure 3a that, while increasing *P* to 100 W, additional peaks were observed in addition to shifting of the starching vibration of Si-O-Si towards a lower frequency with increasing intensity. This alter is credited to two components: expanded vitality input into the plasma framework, and expanded substrate temperature due to RF warming. The vitality input, in this case, RF control, is accepted to have the most noteworthy impact on the properties of a plasma polymer of all statement parameters, as the size of vitality input decides the degree of fracture of the monomer [39]. The two new peaks, such as the broad absorption band centered at 3380 cm^−1^ and 1627 cm^−1^ reveal the presence of hydroxyl groups (OH), starching, and bending vibration of O-H, respectively, most likely associated with Si-OH. The most prominent absorption, 1032 cm^−1^, shifting towards a lower frequency (1000 cm^−1^) with increasing intensity, is likely related to the Si-O stretching vibration in Si-O-Si, in a changed neighboring environment, where Si-CH_2_-Si groups are also involved. The enhanced intensity and broadening of the peak at higher current density pointed to an elongation of the main chain, as well as to a high degree of cross-linking, mainly due to oxygen atoms in Si-O-Si groups. Further evidence of organic groups attached to silicon of the main backbone is provided by the band in the 1352–1373 cm^−1^ range, related to the stretching vibration of methylene groups in Si-CH_2_-Si functional. This feature does not exist either in the monomer or in the case of a film deposition at a lower power regime. Other than the same absorption bands with lower power regime, the low intensity and thin peak appearing around 1260 cm^−1^ reveals symmetric bending vibrations of C-H bonds in methylsilyl (Si(CH_3_)_x_) groups, and the retention of the organic fraction of the HMDSO molecule. This absorption band may have the contribution of methylsilyl (Si(CH_3_)), dimethylsilyl (Si(CH_3_)_2_), and trimethylsilyl (Si(CH_3_)_3_) groups [40], characteristic of organosilicon structures. In addition to this, asymmetric bending vibrations of the same groups are identified by the low-intensity absorption in the range of 1407–1456 cm^−1^, which does not also exist at a lower power regime. The retention of organic fraction from HMDSO is also revealed by the contributions arising at 2955, 2913, and 2877 cm^−1^, ascribed to the stretching mode of C-H in CH_2_ and CH_3_ groups. In the low wavenumber region, the peak at 800 cm^−1^ may arise from contributions of both Si(CH_3_)_2_ bending [37,38] as well as Si-O stretching [41] vibrations. The disappearance of a peak at 845 cm^−1^, caused by the depletion methyl rocking vibration in a Si(CH_3_)_3_ groups, as *P* increased, is indicative of a film with a reduced organic nature. All these findings suggest that the Si-O-Si backbone is surrounded by methyl groups providing a structure that resembles the silicone or slightly oxidized PDMS one, but with a higher crosslinking degree than the conventional polymer. Upon electron collision or argon reaction, the HMDSO molecule can split into a variety of pieces, including (CH_3_)_3_-Si-O-, (CH_3_)_3_-Si-O-Si-O-(CH_3_)_2_, Si-(CH_3_)_3_, and CH_3_. These, in contrast to a large number of other potential species, have a high sticking coefficient, and are capable of heterogeneous gas–surface interactions, which can result in the formation of an organosilicon structure. Another adventitious group, which is not present either in the original HMDSO molecule or in the case of this film at 50 W, is silane (Si-H), detected by the rise of the band around 2170 cm^−1^ [42,43]. The incorporation of this function suggests that the fragments of HMDSO undergo multiple-step reactions in the plasma phase (i.e., the breaking of Si-CH_3_ to detach the –CH_3_ group, then combining with the hydrogen formed from the breaking of C-H bonds) [43], giving rise to species not present in the precursor molecule. Considering such findings, the structure of the film deposited at 100 W is considered to be a crosslinked organosilicon structure, due to the preservation of methyl groups, retaining hydroxyl and silyl groups. That is, with increasing deposition power, the energy and density of activated species growth favor the removal of hydrogen and methyl groups from the monomer fragments, then the deposition of a less content of organics. Removal of organics from the solid phase may also account for such a finding, which could again explain a decreasing contact angle obtained at a moderate plasma excitation power.

Considering the spectrum of the film prepared at 200 W, the main alteration is related to the disappearance of the peaks at 1260 and 3000 cm^−1^, indicating depletion of methylsilyl (Si(CH_3_)_x_) and methyl (CH_3_) groups from the film structure. It indicates an increase in the inorganic character of the material. The displacement of the peak related to Si-O vibration in Si-O-Si groups to higher wavenumber is indicative of structural rarefication [44]. According to Grill and Neumayer [45], the shift of the absorption related to Si-O bonds reveals a change in the SiO_2_ binding angle. For silica films grown under low temperatures, the Si-O absorptions rise at 1060 cm^−1^, while for thermally-grown films, it appears at 1090 cm^−1^ [46]. The shrinkage of the same band reveals increments in the oxidation degree [47]. The substitution of methyl groups by H in the siloxane structures, generating Si-H groups, accounts for the density reduction. The preservation of the peak at 800 cm^−1^, despite the intense loss of methyl groups, suggests this contribution is mainly ascribed to Si-O more than (SiO(CH_3_)_X_) groups. The literature reports [44,48,49,50,51] demonstrate that the density reduction is also associated with an increasing proportion of the tetrahedral oxide (SiO_4_), since it presents higher porosity than the suboxides (SiO_3_, SiO_2,_ and SiO). The presence of SiH is consistent with the presence of suboxides in the film, which can no longer be characterized as an organosilicon material, but as a silicon oxide structure with low proportions of organic contamination, as well as hydroxyl and silyl groups’ inclusion.

Figure 3b shows the infrared spectra of the films prepared under different *P* from HMDSO with TiO_2_ sputtering. When we see the film deposited at 50 W, the main alteration was detected in the spectrum of the film upon the addition of TiO_2_. It showed the appearance of two new pikes corresponding to Si-H (2153 cm^−1^, which is not present either in the monomer HMDSO or in its film corresponding to similar *P*), and Si(CH_3_)_x_ (1411 cm^−1^, bending vibration). Despite the fact that sputtering TiO_2_ through a plasma can enhance the collision of argon and HMDSO, fragmenting the monomer into smaller organic molecules and increasing the concentration of inorganic molecules, we obtain unexpectedly more similar organosilica film, as without TiO_2_ sputtering. This is due to insufficient *P* for sputtering the TiO_2_. This consolation is in agreement with sufficient plasma excitation power, 100 W, a different spectrum is observed quite similar to the spectrum of the films deposited at 200 W without TiO_2_ sputtering. It related to the overall reduction in the band intensities, aside from the disappearance of the methylsilyl bands (2160, 1300–1400 cm^−1^). These two results may be explained in terms of changes in the deposition mechanism caused by the TiO_2_ presence in the process. The recombination of TiO_2_ and HMDSO fragments may provide extra removal of organics, reducing the availability of film precursors as well as generating hybrid Si–O–Ti groups in the film structure. The presence of this function is normally observed by a band of around 993–998 cm^−1^ [52]. The overlapping with the intense contribution attributed to Si–O–Si around 1000 cm^−1^ turns its identification dubious but the contribution of this group to this band is not excluded. The stretching mode of Si–O–Si bonds has been reported in the range of 1010−1150 cm^−1^ [53,54,55,56] the stretching mode of the Ti–O–Si moieties is expected to fall at 1024 cm^−1^ [57]. The low-intensity absorption band around 667 cm^−1^ is also evidence of Ti incorporation, since it represents a Ti–O stretching vibration in Ti–O–Ti [52,58,59,60]. Furthermore, since Ar ions are attracted by the negative self-bias polarization developed at the driven electrode, TiO_2_ fragments are ejected to the plasma phase, accounting for providing precursors for film deposition, but also altering the plasma pressure. The elevation in pressure is proposed to occur with increasing power affecting the deposition mechanisms. Then, the lower intensities of the absorption bands may be a consequence of the deposition rate reduction caused by the TiO_2_ presence in the deposition process. Interestingly, the band related to Si-H was not detected in this spectrum, suggesting a confirmed depletion in the weakly bonded terminal and lateral groups.

Considering the spectrum of the film deposited at a higher power regime, the bands related to organics absorption band in the range of 1351–1363 cm^−1^ and 993–998 cm^−1^, respectively, also showed the methylene groups in Si–CH_2_–Si and the presence of Si–O bond in the Si–O–Si short chain as well in Si–O–Ti short chains. In the contradictory film obtained of a higher power without TiO_2_, the appearance of the peaks at 1260 and 3000 cm^−1^, indicating restoration of methylsilyl (Si(CH_3_)_x_) and methyl (CH_3_) groups in the film structure, then increasing the organic character of the material. This is due to the fact that an excess of titanium dioxide powder is sprayed into the plasma reaction chamber.

### 3.3. Deconvolution of SiO IR Bands

**Figure 4:**
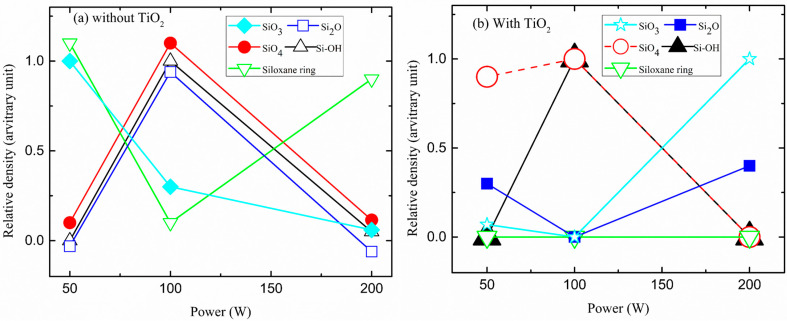
The relative density of Si-OH, Si_2_O, siloxane ring, and SiOx groups in the film deposition, as a function of plasma excitation of power, of (**a**) HMDSO and (**b**), HMDSO with TiO_2_ sputtering.

The Si-O stretching band was deconvoluted into several disparate peaks: 1123 ± 21, 1067 ± 17, 1016 ± 3, 995 ± 5, and 935 ± 1 cm^−1^, to see additional structural information upon the combined effect of *P* and TiO_2_, and the result is shown in Figure 4. The peak at 1123 ± 21 cm^−1^ is interpreted as a short-chain Si-O-Si, as a cage-type structure [61,62,63], where the Si atom is bonded to four O atoms at an angle of 150° [38,45]. Only the deposited film at 50 W, does not show this cage-type structure. The peak at 1067 ± 17 cm^−1^ is assonated to a long-chain Si-O-Si terminated with methyl or hydroxyl groups [61,63]. However, sometimes this area of the spectrum is viewed as only one peak of around 1050–1063 cm^−1^, which is then assigned to siloxane networks with Si-O-Si angles of ~144° [16,44]. It is the only structure that was shown in all deposited films. The peak at 1016 ± 3 cm^−1^ is ascribed to the siloxane ring (Si-O-Si in cyclotrisiloxanes) [61,62] and 995 ± 5 cm^−1^ is for Si suboxides (Si_2_O, Si bound with two oxygen atoms forming smaller Si-O-Si angles < 144) (62). Only the films deposited at 100 and 200 W did not show this structure. The peak at the lowest wavenumber, around ~935 cm^−1^, is usually referred to as the Si-OH silicon-hydroxyl stretching [64]. Only the deposited film at lower *P* does not show this silicon-hydroxyl structure. It was also confirmed that the absence of stretching and bending vibration of the O-H group in this *P* regime. An estimate of the trend in the density of these groups was obtained by the integrated absorption method [65] using the deconvolution components. In general, there is a tendency for the relative density of siloxane networks to decrease, and suboxides and stoichiometric oxide to increase, in a complementary way, confirming the conversion of one type of structure to another when increasing plasma excitation *P*.

### 3.4. Roughness

**Figure 5:**
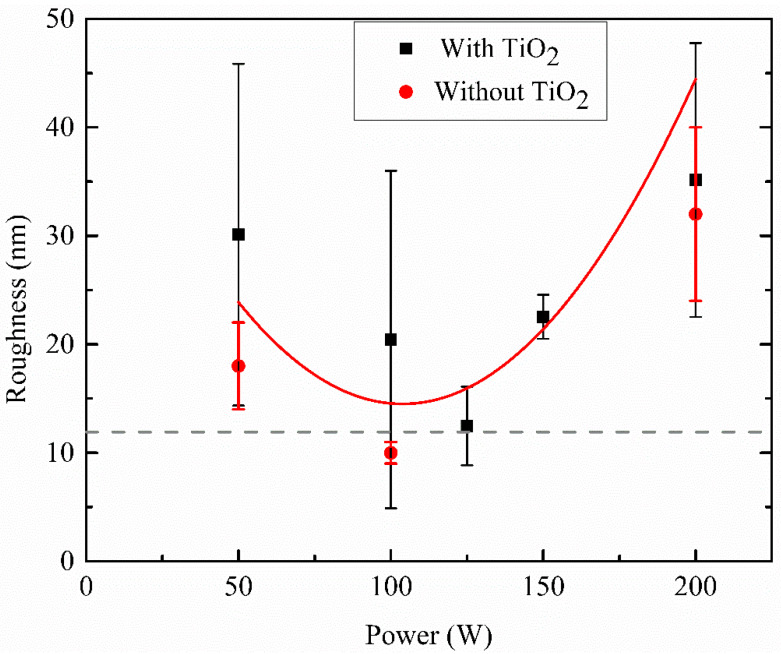
The roughness as a function of the applied power of films deposited in discharges composed of HMDSO and argon, with and without TiO_2_. The dotted line represents the roughness of the clean glass substrate.

**Figure 6:**
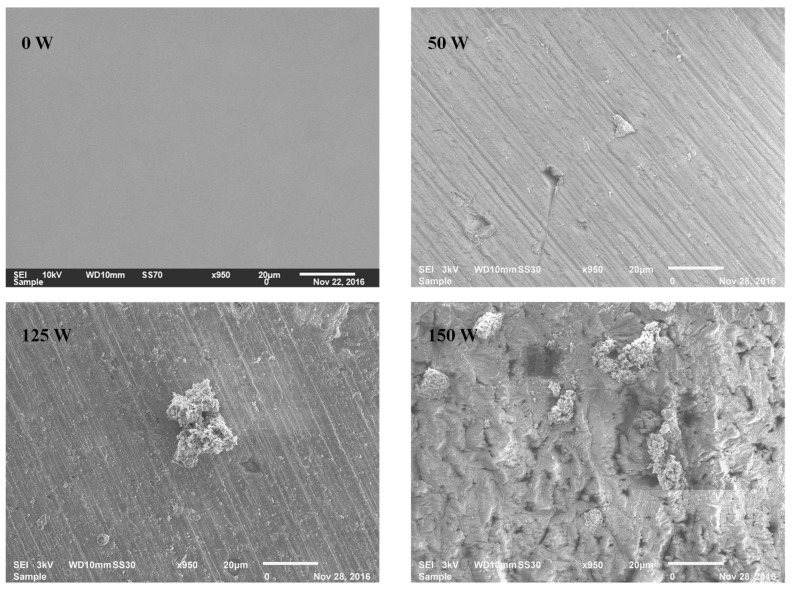
Scanning electron micrographs of aluminum substrates as-received and coated in HMDSO and TiO_2_ containing discharges at different power.

Figure 5 shows the average *R_a_* roughness of HMDSO films as a function of *P*. The dashed line in this figure represents the value of the roughness of a clean glass surface exposed to the monomer without plasma ignition. Although the surface becomes rougher after the deposition takes place, with the rise of power until 125 W, it continuously decreased until reaching pure glass roughness. Afterwards, it increased again with a further increase in *P*. This is due to the creation of irregularities on the surface by the sputtering of deposited material and the incorporation of TiO_2_ particles, as observed in Figure 6. This significant effect of sputtering plasma also increases surface cracking in magnitude, which may be due to the incorporation of functionalities in the surface.

### 3.5. Morphology

Figure 6 shows the scanning electron microscopy (SEI) micrographs of films deposited on aluminum substrates. As can be observed, the morphology of the aluminum surface was changed significantly upon film deposition, depending on plasma excitation *P*. Without sputtering, the smoothness and uniformity are increased with *P* until 100 W and, thereafter, one may observe small crystal-like particles. This is associated with the small thickness of the film deposited at a higher *P*. In addition to this, a film produced at 150 W shows aggregate incorporation of TiO_2_ spongy-shaped particles uniformly distributed on the whole evaluated part of the substrate. Particles are also observed in a small part of the surface on the film deposited at 125 W. This micrograph corroborates very well with previously reported Si-doped TiO_2_ nanocrystals and TiO_2_ nanoparticles synthesized by the sol-gel technique [58].

**Figure 7:**
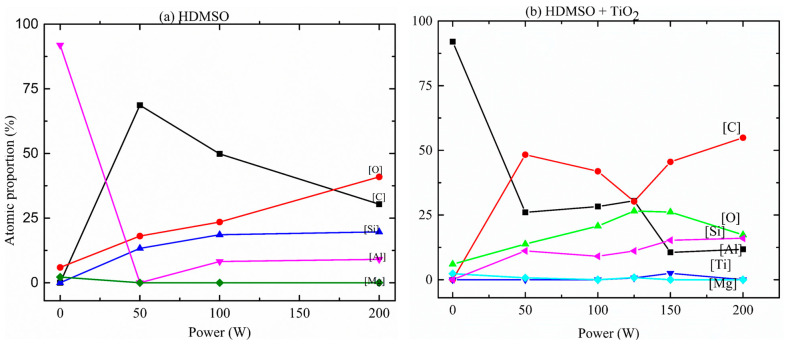
The atomic proportion of carbon, titanium, silicon, and oxygen in the films as a function of the plasma excitation power, (**a**) without TiO_2_ sputtering, (**b**) with TiO_2_ sputtering.

Figure 7 shows the atomic proportions (%at.) of magnesium [Mg], carbon [C], oxygen [O], titanium [Ti], aluminum [Al], and silicon [Si] in films derived from the EDS spectra as a function of excitation *P*. Uncoated polished Al elemental analysis revealed that the substrate contains 92% Al, 6% O, and 2% Mg. It has also been observed that the film deposited at a lower power regime contains 69% C, 18% Si, and 13% O. These proportions are coherent with the proportions of the (C_4_H_x_SiO)n polymer chemical formula, which is 68% C, 16% Si, and 16% O, as H atoms are not assessed by EDS, and considering the O_2_ content of the substrate. It indicates that, at a lower power regime, unfragmented HMDSO monomer may also have been deposited.

Considering the film deposited at a moderate *P* regime, 8% of Al was observed, in addition to a decreasing carbon proportion from 69% to 50%. This is due to the beam penetration being larger than the film thickness, in agreement with decreasing thickness with *P*. It is also coherent with the proportions of C (50%), O (25%), and Si (25%) present in the chemical formulae of the polydimethylsiloxane (C_2_H_6_SiO)n, when H atoms are not taken into account. Further, silicon and oxygen atomic proportions increased, which indicates that, in this regime, a more fragmented monomer was deposited.

For the highest power regime, 200 W, significant opposite variations were observed in [C] and [O], while [Si] remained roughly constant. It is important to note, however, that only an increase in *P* provoked a decrease in [C], changing the overall film stoichiometry once the atomic percentage was normalized at 100%. In a general way, even though the EDS spectra of films prepared at 50, 100, and 200 W from HMDSO and Ar atmosphere with TiO_2_ sputtering do not contain Ti, their atomic proportion and structures are slightly different in comparison with the films obtained from HMDSO and Ar atmosphere with similar plasma excitation power.

### 3.6. Contact Angle and Surface Energy

**Figure 8:**
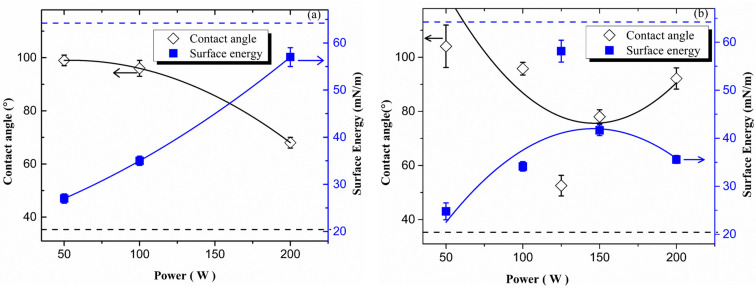
The contact angle and surface energy of the films derived from, (**a**) HMDSO and (**b**) HMDSO, with TiO_2_ plasmas as a function of plasma excitation power.

The water contact angle (WCA) and the surface energy of the films derived from HMDSO and HMDSO with TiO_2_ sputtering were measured as a function of *P*, and the results are shown in Figure 8. As can be seen in this figure, films with different wettability were obtained, depending on plasma deposition condition. Although, for HMDSO deposition using the lower and moderate *P*, hydrophobic (θ > 95°) coatings have been produced, the WCA continuously decreased with *P*. It is interesting to note that silanol (hydroxyl), and silane (Si-H), groups were detected in the infrared spectra (1627 and 3380 cm^−1^) and (2170 cm^−1^), respectively, at 100 W. The presence of such species is normally associated with points where hydrolyzation can occur upon exposition to the atmosphere or water solutions, providing an early deterioration of the barrier properties. On the other hand, by further increasing *P*, up to 200 W, there is a significant variation in θ, resulting in hydrophilic films with a contact angle around 68°. It is interesting to note that the FTIR spectrum of such film revealed the absence of the peaks at 1260 and 3000 cm^−1^, indicating depletion of methylsilyl (Si(CH_3_)_x_) and methyl (CH_3_) groups from the film structure, then increasing the inorganic character of the film.

Figure 8b shows the contact angle, θ, and surface energy of glass (dotted line), and the films prepared by PECVD under different plasma excitation power, *P*, from HMDSO and Ar atmospheres together with TiO_2_ sputtering. Films with different wettability were obtained, depending on the *P*. For deposition using the lower excitation *P*, the resulting film is hydrophobic with contact angles above 104°, which is in close agreement with the water contact angle of SiO_x_C_y_H_z_ deposited from pure HMDSO plasmas [33], and more hydrophobic than the film obtained from HMDSO and Ar atmospheres with a similar *P*. It is interesting to observe a difference in wettability at the similar lower *P* regime, due to the higher thickness of a film obtained from HMDSO and Ar atmospheres together with TiO_2_ sputtering. Even though films prepared from HMDSO, Ar atmosphere together with TiO_2_ plasmas present additional Si-H groups, they are shielded/surrounded by high concentration non-polar methyl groups, reducing the electrostatic force over water molecules. Therefore, the higher the proportion of trimethylsilyl on the surface, the higher the contact angle, in agreement with the increasing intensity of IR bands at 3000 and 800 cm^−1^. Increasing *P* to 100 W, the wettability of the film decreased to around 96°. It is also, still hydrophobic and equivalent contact angle value to the film obtained from HMDSO and Ar atmosphere at 100 W.

When *P* > 100 W, it is observed a sudden decrease of film water contact angle results in hydrophilic surfaces (θ < 90°), in the case of 125 W. It is intriguing to note the drastic change in water wettability when HMDSO, Ar, and TiO_2_ plasma films are produced at 125 W. The large concentration of polar Si-O and Ti-O groups initially gives the material a high surface energy, which favors the inclusion of O-H atmospheric groups. These groups facilitate hydrogen bonding between atmospheric water molecules, which influences the way water distributes over the surface. Another interesting aspect is the super-hydrophilicity effect of TiO_2_ incorporation on thin films. The wettability of silica-like film can be further increased by increasing the surface polarization, which will create the possibility of the formation of more hydrogen bonds between the water and the film. This was performed by incorporating Ti-O bonds in the film, which have comparatively higher differences in electronegativity than Si-O. Therefore, the contact angle of a film from HMDSO, Ar atmosphere together with TiO_2_ sputtering at 125 W decreased the water contact angle of silica film (65°) to 52°. However, the water contact angle for TiO_2_ films deposited from titanium isopropoxide and oxygen plasma was around 24°, as revealed by previous studies. The presence of both Ti-O-Ti and Ti-OH bonds contributed to the hydrophobicity. These results were expected, since the EDS and the FTIR confirmed the presence of a larger inorganic character of coatings containing titanium (only at 125 W and 150 W). Although the amount of Ti at 125 W is less than that of 150 W, the contact angle of 125 W is lower, closer to the clean glass contact angle. This may be due to the higher thickness of the film deposited at 150 W than at 125 W (Figure 2).

The films obtained from HMDSO, Ar atmosphere together with TiO_2_, when the applied power was 200 W, were hydrophobic. In addition to significant differences of films obtained from HMDSO, Ar atmosphere without TiO_2_ with the same corresponding *P*., it is interesting that the spectrum of the film without TiO_2_ shows no peaks at 1260 and 3000 cm^−1^, indicating depletion of methylsilyl (Si(CH_3_)_x_) and methyl (CH_3_) groups, then increasing the inorganic character of the film. On the other hand, the spectrum of the film with TiO_2_ deposition presents peaks around 1260 and 3000 cm^−1^, indicating methylsilyl (Si(CH_3_)_x_) and methyl (CH_3_) groups in the film structure. It implies that the surface energy of the thin film deposition was shown the opposite trend of the water contact angle, and it is lower than the surface energy of the glass before thin film deposition.

## 4. Conclusions

In this work, hydrophilic, physically stable, and relatively thick a-C: H: Si: O: Ti films as well hydrophobic, C: H: Si: O: films were deposited by PECVD from HMDSO/argon plasma co-sputtering, with and without powder titanium dioxide, on polished aluminum, glass, and carbon steel substrates. It has been demonstrated that the plasma excitation power influences the properties of films. As the RF power was increased, the deposition rates, water contact angle, thickness, and roughness were firstly decreased and then drastically increased as an effect of TiO_2_ powder, in addition to plasma excitation power.

Even though titanium has not been detected in the film, the deposition with TiO_2_ at 50 and 100 W resulted in rougher coatings and the lowest surface energy when compared to plasma deposition without TiO_2_ powder at the same plasma excitation power, indicating that a spray of powder titanium dioxide through the plasma reaction chamber alters internal pressure and reduces the net excitation energy of the plasma. The increase in power also affects the degree of fragmentation of HMDSO, resulting on the formation of several smaller fragments of organic compounds, which are withdrawn by the vacuum system instead of deposition to the substrate.

## Figures and Tables

**Table 1:** Wavenumbers and assignments of the bands detected in the infrared spectra of the film deposited with 20% HMDSO, with and without TiO_2_ in the feed. The symbols ν, and δ stand for stretching and deformation vibrations, respectively.

Wavenumber (cm^−1^)	Assignments
481–484, 990	δ Ti-O, Ti-O-Si bond
759–766	δ Si-C
794–810	ν CH_3_
899–981, 2215–2238	ν H-Si
1011–1037	ν SiO
1253–1255	δ CH_3_
1446–1459, 1400–1413	δ CH
1351–1363	δ CH_2_
1607–1623	δOH
2941–2961	ν_a_CH
3349–3477	ν OH

## Data Availability

The data presented in this study are available on request from the corresponding author.
